# Newer antidepressant for Japanese adults with major depressive disorder: A systematic review and meta‐analysis

**DOI:** 10.1002/npr2.12376

**Published:** 2023-08-30

**Authors:** Taro Kishi, Kenji Sakuma, Masakazu Hatano, Yuki Matsuda, Satoru Esumi, Nobumi Miyake, Itaru Miura, Hikaru Hori, Masaki Kato, Nakao Iwata

**Affiliations:** ^1^ Department of Psychiatry Fujita Health University School of Medicine Toyoake Aichi Japan; ^2^ Department of Pharmacotherapeutics and Informatics Fujita Health University School of Medicine Toyoake Aichi Japan; ^3^ Department of Psychiatry Jikei University School of Medicine, Minato Tokyo Japan; ^4^ Faculty of Pharmaceutical Sciences Kobe Gakuin University Kobe Hyogo Japan; ^5^ Department of Neuropsychiatry St. Marianna University School of Medicine Kawasaki Kanagawa Japan; ^6^ Department of Neuropsychiatry Fukushima Medical University School of Medicine Fukushima Japan; ^7^ Department of Psychiatry, Faculty of Medicine Fukuoka University Fukuoka Japan; ^8^ Department of Neuropsychiatry Kansai Medical University Osaka Japan

**Keywords:** efficacy, Japanese major depressive disorder, newer antidepressant, safety, systematic review and meta‐analysis

## Abstract

**Introduction:**

The question remains to be elucidated: “Is treatment with antidepressants at doses approved in Japan effective for Japanese patients with MDD?” It is crucial to confirm this in order to provide appropriate treatments for Japanese patients with major depressive disorder (MDD). Therefore, we conducted a systematic review and random‐effects pairwise meta‐analysis including these nine double‐blind, randomized, placebo‐controlled trials.

**Methods:**

We calculated the standardized mean difference (SMD) and risk ratio (RR) with a 95% confidence interval (95% CI).

**Results:**

Pooled newer antidepressants outperformed placebo regarding improvement of depressive symptom scale scores [SMD (95% CI) = −0.20 (−0.27, −0.12), *p* < 0.00001], response to treatment [RR (95% CI) = 1.23 (1.13, 1.32), *p* < 0.00001], and remission rate [RR (95% CI) = 1.30 (1.16, 1.45), *p* < 0.00001]. Although all‐cause discontinuation was not significantly different between the treatment groups, the pooled antidepressant group showed a higher discontinuation rate due to adverse event [RR (95% CI) = 1.60 (1.13, 2.26), *p* = 0.007] and a higher incidence of at least one adverse event than the placebo group [RR (95% CI) = 1.13 (1.08, 1.18), *p* < 0.00001].

**Discussion:**

We concluded that newer antidepressants are effective for Japanese adults with MDD although the clinicians must monitor the health conditions of these individuals.

## Abstract 2

Results of the meta‐analysis. (A) The improvement of depressive symptoms. (B) Response to treatment. (C) Remission rate. (D) All‐cause discontinuation. 95% CI, 95% confidence interval; DUL, duloxetine; ESC, escitalopram; IV, inverse variance; M‐H, Mantel–Haenszel; MIR, mirtazapine; PAR‐CR (‐IR), paroxetine‐controlled‐release (‐immediate release); Std. Mean Difference, standardized mean difference; VEN, venlafaxine; VOR, vortioxetine.

## Graphical abstract

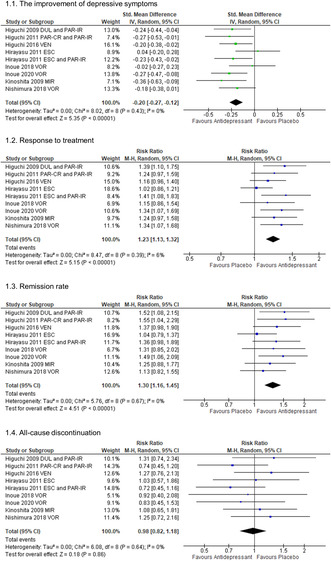

## 1 INTRODUCTION

The Japanese Society of Mood Disorders released a treatment guideline for adults with major depressive disorder (MDD): “there is no doubt that antidepressants are effective in treating moderate to severe MDD.”^1^ This is based on review articles on antidepressant trials conducted in countries other than Japan.^2^, ^3^ However, due to the differences in the approved dose of antidepressant between Japan and other countries such as the United States (Table S1), following the principles of evidence‐based medicine, it might be unsuitable to propose a treatment for Japanese with MDD based on the results of clinical trials outside of Japan.^4^ To date, there are nine double‐blind, randomized, placebo‐controlled trials (DBRPCTs) aimed at determining the efficacy outcomes of antidepressant in Japan (Table S2).^5^, ^6^, ^7^, ^8^, ^9^, ^10^, ^11^, ^12^, ^13^ However, the results from these trials were inconsistent (Table S3). Thus, the question remains to be elucidated: “Is treatment with antidepressants at doses approved in Japan effective for Japanese patients with MDD?” It is crucial to confirm this in order to provide appropriate treatments for Japanese patients with MDD. Therefore, we conducted a systematic review and random‐effects pairwise meta‐analysis^14^ including these nine DBRPCTs.

## 2 METHODS

This study was performed in accordance with the Preferred Reporting Items for Systematic Reviews and Meta‐Analyses guidelines (Table S4).^15^ At least two authors simultaneously and independently conducted the literature search, data extraction, and data entry. Furthermore, the authors double‐checked all data for accuracy. The detailed methods are presented in the Open Science Framework (https://osf.io/h6c2e). The PICO strategy of our study was as follows:

P: Japanese adults with MDD.
I: Antidepressant.
C: Placebo.
O: Improvement of depressive symptom scale scores (primary outcome), response to treatment, remission rate, all‐cause discontinuation, discontinuation due to adverse event, and at least one adverse event (data synthesis for efficacy outcomes: Table S3).

The following studies were excluded: (1) studies focusing on specific generations (e.g., children and/or adolescents or older individuals), (2) studies including individuals with the following characteristics; MDD with psychotic features, MDD with severe somatic illness, treatment resistant depression, and/or bipolar disorder, and (3) studies that allowed to use an antipsychotic during the study.^16^ We searched the PubMed, the Cochrane Library, ICHUSHI, and Embase databases for studies published before May 3, 2023, without language restriction. The search terms for PubMed, the Cochrane Library, and ICHUSHI (Japanese) included (fluvoxamine OR paroxetine OR sertraline OR escitalopram OR milnacipran OR duloxetine OR venlafaxine OR mirtazapine OR vortioxetine) AND (placebo) AND (Japan OR Japanese) AND (depressi*). The search terms for Embase included (‘randomized controlled trial’/exp OR ‘randomized controlled trial’) AND (‘major depression’/exp OR ‘major depression’) AND (‘placebo’/exp OR ‘placebo’) AND (‘japanese (people)’/exp OR ‘japanese (people)’). Additionally, reference lists of the included articles were manually searched for additional relevant published and unpublished research, including conference abstracts. We also searched clinical trial registries (ClinicalTrials.gov [http://clinicaltrials.gov/] and the World Health Organization International Clinical Trials Registry Platform [http://www.who.int/ictrp/search/en/]) to ensure the RCTs were comprehensive and to minimize the effect of publication bias. Moreover, we also used the drug package insert for each newer antidepressant to determine search criteria.^17^ Any discrepancies in the selected articles were resolved by consensus of the authors. If multiple papers or academic conference abstracts were reported despite the same research, the literature was screened by confirming the clinical trial registration number and/or reference to past review articles.

The authors independently extracted data from all the included studies. All analyses were based on the intention‐to‐treat or modified intention‐to‐treat principles. When the data required for the meta‐analysis were incomplete, we contacted the original study investigators to obtain the unpublished data. We also searched for missing data in published systematic review articles.

We calculated the standardized mean difference (SMD) and risk ratio (RR) with a 95% confidence interval (95% CI) using a random‐effects model and *I* ^2^ statistics (*I* ^2^ ≥ 50% indicating heterogeneity).^4^ The pairwise meta‐analysis was conducted using the Review Manager software (version 5.4).^4^ In case of significant differences in the dichotomous variable between the treatment groups, the number needed to treat to benefit (NNTB) or harm (NNTH) was estimated using the exact rates and 95%CIs in each outcome in both the antidepressant and placebo groups estimated by a single‐group summary meta‐analysis. Egger's regression was also conducted to detect publication bias in the meta‐analysis. These analyses were conducted using the Comprehensive Meta‐Analysis Software Version 3 (Biostat Inc.).

## 3 RESULTS

**FIGURE 1:**
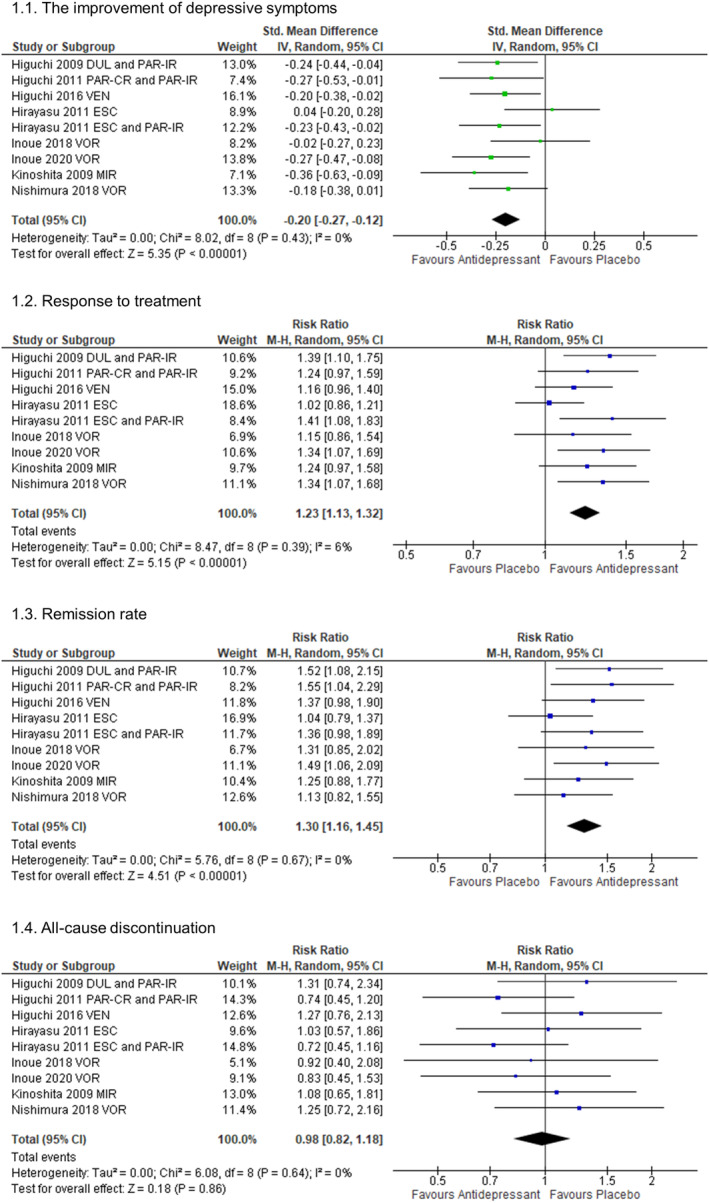
Results of the meta‐analysis. (A) The improvement of depressive symptoms. (B) Response to treatment. (C) Remission rate. (D) All‐cause discontinuation. 95% CI, 95% confidence interval; DUL, duloxetine; ESC, escitalopram; IV, inverse variance; M‐H, Mantel–Haenszel; MIR, mirtazapine; PAR‐CR (‐IR), paroxetine‐controlled‐release (‐immediate release); Std. Mean Difference, standardized mean difference; VEN, venlafaxine; VOR, vortioxetine.

The result of the literature search is shown in Figure S1. Nine DBRPCTs were included in our systematic review (Table S1). In all trials, the participants were diagnosed with moderate to severe MDD (Table S2), a newer antidepressant (duloxetine, escitalopram, mirtazapine, paroxetine, venlafaxine, and vortioxetine) was used. No studies have a high risk of bias in the analysis using the Risk of Bias 2 tool (Figure S2).^4^ Pooled newer antidepressants outperformed placebo regarding improvement of depressive symptom scale scores [SMD (95% CI) = −0.20 (−0.27, −0.12), *p* < 0.00001, *I* ^2^ = 0%, Figure 1], response to treatment [RR (95% CI) = 1.23 (1.13, 1.32), *p* < 0.00001, *I* ^2^ = 6%, NNTB (95% CI) = 9 (7, 16), Figure 1], and remission rate [RR (95% CI) = 1.30 (1.16, 1.45), *p* < 0.00001, *I* ^2^ = 0%, NNTB (95% CI) = 8 (12, 22), Figure 1]. No publication bias for the primary outcome was detected (Figure S3). Although all‐cause discontinuation was not significantly different between the treatment groups (Figure 1), the pooled antidepressant group showed a higher discontinuation rate due to adverse event [RR (95% CI) = 1.60 (1.13, 2.26), *p* = 0.007, *I* ^2^ = 0%, NNTH (95% CI) = 56 (31, 143), Figure S4] and a higher incidence of at least one adverse event than the placebo group [RR (95% CI) = 1.13 (1.08, 1.18), *p* < 0.00001, *I* ^2^ = 0%, NNTH = not significant, Figure S5].

## 4 DISCUSSION

Our meta‐analysis demonstrated that the pooled newer antidepressants group was superior to the placebo group in terms of all efficacy outcomes without considerable heterogeneity. However, the pooled newer antidepressants seemed to have worse tolerability than placebo. We concluded that newer antidepressants are effective for Japanese adults with MDD although the clinicians must monitor the health conditions of these individuals.

Although the effect size of improvement in depressive symptom scale scores (continuous variable) was small, that of response to treatment (dichotomous variable) was moderate. Thus, there might be a clear difference between Japanese patients with MDD that substantially responded to an antidepressant and those who did not respond well to the antidepressant. Due to possible differences in the efficacy, acceptability, tolerability, and safety of antidepressants in Japanese patients with MDD similar to other population,^18^ a network meta‐analysis including only Japanese population should be conducted.

Our study has some limitations. First, our meta‐analysis did not include newer antidepressants such as fluvoxamine, sertraline, and milnacipran because no DBRPCTs for these were conducted in Japan. Second, some DBRPCTs included both Japanese and other populations (Table S3). However, although we conducted a subgroup meta‐analysis excluding the studies for the primary outcome, pooled newer antidepressants also outperformed placebo regarding improvement of depressive symptom scale scores [SMD (95% CI) = −0.19 (−0.28, −0.10), *p* < 0.0001, *I* ^2^ = 22%].

## AUTHOR CONTRIBUTIONS

TK had full access to all data and took full responsibility for the data integrity and the accuracy of the data analysis. TK developed the study concept and design and performed the statistical analyses. All authors interpreted the data and wrote the manuscript. NI supervised the review.

## FUNDING INFORMATION

Grant‐in‐Aid for Scientific Research (C) (23 K06998).

## CONFLICT OF INTEREST STATEMENT

The authors have no specific conflicts of interest to declare concerning this study. They would like to disclose the following interests that have arisen in the last 3 years: TK has received speaker's honoraria from Eisai, Janssen, Meiji, MSD, Otsuka, Sumitomo, Takeda, Tanabe‐Mitsubishi, and Viatris and research grants from Eisai, Grant‐in‐Aid for Scientific Research (C) (19K08082 and 23K06998), Japan Agency for Medical Research and Development (JP22dk0307107, JP22wm0525024, JP23dk0307117, JP23wm0525024, and JP23dk0307122), and the Japanese Ministry of Health, Labour and Welfare (21GC1018). KS has received speaker's honoraria from Eisai, Janssen, Kyowa, Meiji, Otsuka, Sumitomo, and Takeda and research grants from Grant‐in‐Aid for Young Scientists (19K17099), Grant‐in‐Aid for Scientific Research (C) (23 K06998), Fujita Health University School of Medicine Research Grant for Early‐Career Scientists, and Japan Agency for Medical Research and Development (JP22dk0307107). MH has received speaker's honoraria from Meiji, Sumitomo, and WELCIA, and Grant‐in‐Aid for Early‐Career Scientists (23K14827). YM has received speaker's honoraria from, Otsuka, Sumitomo, Takeda, Teijin, Lundbeck, and Viatris. SE has received speaker's honoraria from Eisai, Otsuka, and research grants from Grant‐in‐Aid for Scientific Research (C) (23K06255). NM has received speaker's honoraria from Meiji, Otsuka, Sumitomo, Takeda, and Viatris. IM has received speaker's honoraria from Eisai, Janssen, Meiji, Otsuka, Sumitomo, Takeda, Tanabe‐Mitsubishi, Towa, and Yoshitomi, and Grant‐in‐Aid for Scientific Research (B) (21H02133) and (C) (17K10279 and 23K07038). HH has received speaker's honoraria from Eisai, Janssen, Meiji, Otsuka, Sumitomo, Takeda, and Viatris and Grant‐in‐Aid for Scientific Research (C) (23K09705). M.K. received consulting fees from Sumitomo, Otsuka, Lundbeck, Takeda, and Shionogi; and payment/honoraria from Sumitomo, Otsuka, Meiji, Eli Lilly, MSD, Pfizer, Janssen, Shionogi, Mitsubishi Tanabe, Takeda, Lundbeck, Viatris, Eisai, Kyowa, and Ono and has received grant funding from the Japan Society for the Promotion of Science (22 K07607), Japan Agency for Medical Research and Development (JP20dk0307081), SENSHIN Medical Research Foundation, the Japan Research Foundation for Clinical Pharmacology and the Japanese Society of Clinical Neuropsychopharmacology. NI has received speaker's honoraria from Eisai, Janssen, Meiji, Otsuka, Sumitomo, Takeda, Tanabe‐Mitsubishi, and Viatris and research grants from Daiichi Sankyo, Eisai, Meiji, Otsuka, Sumitomo, Takeda, and Tanabe‐Mitsubishi.

## ETHICS STATEMENT

Approval of the research protocol by an Institutional Reviewer Board: N/A.

Informed Consent: N/A.

Registry and the Registration No. of the study/trial: N/A.

Animal Studies: N/A.

## Supporting information


Appendix S1

## Data Availability

The most descriptive variables are openly available in the articles of the studies that cited in this paper. Additional variables can be shared upon request to the corresponding author upon agreement of the original investigators and pharmaceutical companies who shared data confidentially, unless the proposed analysis competes with future research of the corresponding author's team.
