# Supplementary material for: Newer antidepressant for Japanese adults with major depressive disorder: A systematic review and meta‐analysis
Source: Neuropsychopharmacol Rep. 2023 Aug 30;44(1):216–20. doi: 10.1002/npr2.12376 (PMC10932784; doi:10.1002/npr2.12376)
Supplement: Supplementary file 1 — Appendix S1 [file NPR2-44-216-s001.docx]

**Figure S1. Preferred Reporting Items for Systematic reviews and Meta-Analyses flow diagram**

**
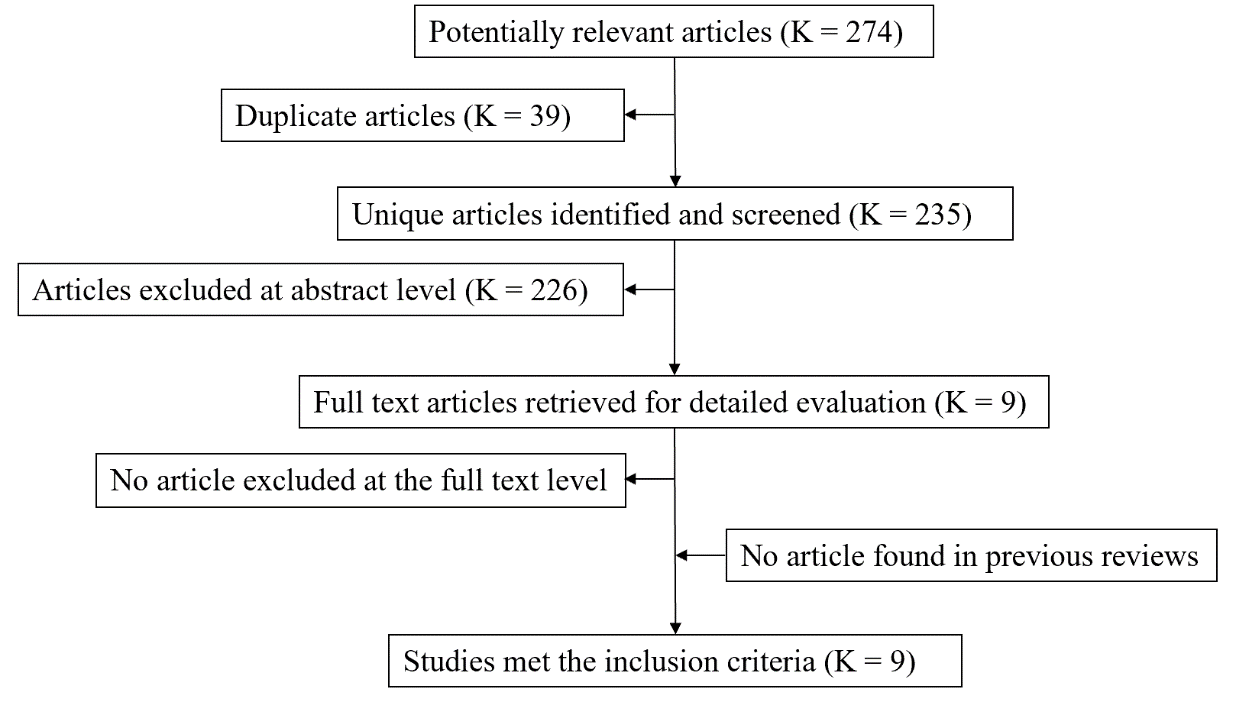
**

Review articles that we read for the literature search

1. Cipriani A, Furukawa TA, Salanti G et al. Comparative efficacy and acceptability of 21 antidepressant drugs for the acute treatment of adults with major depressive disorder: a systematic review and network meta-analysis. *Lancet* 2018; **391**: 1357-1366.

2. Cipriani A, Koesters M, Furukawa TA et al. Duloxetine versus other anti-depressive agents for depression. *Cochrane Database Syst Rev* 2012; **10**: CD006533.

3. Cipriani A, La Ferla T, Furukawa TA et al. Sertraline versus other antidepressive agents for depression. *Cochrane Database Syst Rev* 2010: CD006117.

4. Cipriani A, Santilli C, Furukawa TA et al. Escitalopram versus other antidepressive agents for depression. *Cochrane Database Syst Rev* 2009: CD006532.

5. Cipriani A, Signoretti A, Furukawa TA et al. Venlafaxine versus other anti-depressive agents for depression. *Cochrane Database Syst Rev* 2007.

6. Koesters M, Ostuzzi G, Guaiana G, Breilmann J, Barbui C. Vortioxetine for depression in adults. *Cochrane Database Syst Rev* 2017; **7**: CD011520.

7. Nakagawa A, Watanabe N, Omori IM et al. Milnacipran versus other antidepressive agents for depression. *Cochrane Database Syst Rev* 2009: CD006529.

8. Omori IM, Watanabe N, Nakagawa A et al. Fluvoxamine versus other anti-depressive agents for depression. *Cochrane Database Syst Rev* 2010: CD006114.

9. Purgato M, Papola D, Gastaldon C et al. Paroxetine versus other anti-depressive agents for depression. *Cochrane Database Syst Rev* 2014; **2014**: CD006531.

10. Watanabe N, Omori IM, Nakagawa A et al. Mirtazapine versus other antidepressive agents for depression. *Cochrane Database Syst Rev* 2011: CD006528.

Articles included in our systematic review

1. Higuchi T, Hong JP, Jung HY, Watanabe Y, Kunitomi T, Kamijima K. Paroxetine controlled-release formulation in the treatment of major depressive disorder: a randomized, double-blind, placebo-controlled study in Japan and Korea. Psychiatry Clin Neurosci 2011; 65: 655-63.

2. Higuchi T, Kamijima K, Nakagome K et al. A randomized, double-blinded, placebo-controlled study to evaluate the efficacy and safety of venlafaxine extended release and a long-term extension study for patients with major depressive disorder in Japan. Int Clin Psychopharmacol 2016; 31: 8-19.

3. Higuchi T, Murasaki M, Kamijima K. Clinical evaluation of duloxetine in the treatment of major depressive disorder−Placebo- and paroxetine-controlled double-blind comparative study−. Jpn. J. Clin. Psychopharmacol. 2009; 12: 1613-1634.

4. Hirayasu Y. A dose-response and non-inferiority study evaluating the efficacy and safety of escitalopram in patients with major depressive disorder: a placebo- and paroxetine- controlled, double-blind, comparative study. Jpn. J. Clin. Psychopharmacol. 2011; 14: 883-899.

5. Hirayasu Y. A dose-response study of escitalopram in patients with major depressive disorder: a placebo-controlled, double-blind study. Jpn. J. Clin. Psychopharmacol. 2011; 14: 871-882.

6. Inoue T, Nishimura A, Sasai K, Kitagawa T. Randomized, 8-week, double-blind, placebo-controlled trial of vortioxetine in Japanese adults with major depressive disorder, followed by a 52-week open-label extension trial. Psychiatry Clin Neurosci 2018; 72: 103-115.

7. Inoue T, Sasai K, Kitagawa T, Nishimura A, Inada I. Randomized, double-blind, placebo-controlled study to assess the efficacy and safety of vortioxetine in Japanese patients with major depressive disorder. Psychiatry Clin Neurosci 2020; 74: 140-148.

8. Kinoshita T. A double-blind, placebo-controlled study of a new antidepressant, mirtazapine, in depressed patients. Jpn. J. Clin. Psychopharmacol. 2009; 12: 289-306.

9. Nishimura A, Aritomi Y, Sasai K, Kitagawa T, Mahableshwarkar AR. Randomized, double-blind, placebo-controlled 8-week trial of the efficacy, safety, and tolerability of 5, 10, and 20 mg/day vortioxetine in adults with major depressive disorder. Psychiatry Clin Neurosci 2018; 72: 64-72.

**Figure S2. Risk of bias summary**

|  | Randomization process | Deviation from intended intervention | Missing outcome data | Measurement of the outcome | Selection of the reported result | Overall risk of bias |
| --- | --- | --- | --- | --- | --- | --- |
| Higuchi 2009 DUL vs PAR-IR vs PLA | Some concerns | Some concerns | Low risk | Low risk | Low risk | Some concerns |
| Higuchi 2011 PAR-CR vs PAR-IR vs PLA | Some concerns | Some concerns | Low risk | Low risk | Low risk | Some concerns |
| Higuchi 2016 VEN vs PLA | Some concerns | Some concerns | Low risk | Low risk | Low risk | Some concerns |
| Hirayasu 2011 ESC vs PAR-IR vs PLA | Some concerns | Some concerns | Low risk | Low risk | Low risk | Some concerns |
| Hirayasu 2011 ESC vs PLA | Some concerns | Some concerns | Low risk | Low risk | Low risk | Some concerns |
| Inoue 2018 VOR vs PLA | Some concerns | Some concerns | Low risk | Low risk | Low risk | Some concerns |
| Inoue 2020 VOR vs PLA | Low risk | Some concerns | Low risk | Low risk | Low risk | Some concerns |
| Kinoshita 2009 MIR vs PLA | Some concerns | Some concerns | Low risk | Low risk | Low risk | Some concerns |
| Nishimura 2018 VOR vs PLA | Some concerns | Some concerns | Low risk | Low risk | Low risk | Some concerns |

Version 2 of the Cochrane risk-of-bias tool for randomized trials (https://www.riskofbias.info/)

DUL: duloxetine, ESC: escitalopram, MIR: mirtazapine, PAR-CR (-IR): paroxetine-controlled-release (-immediate release), VEN: venlafaxine, VOR: vortioxetine

**Figure S3. Funnel plot**

**
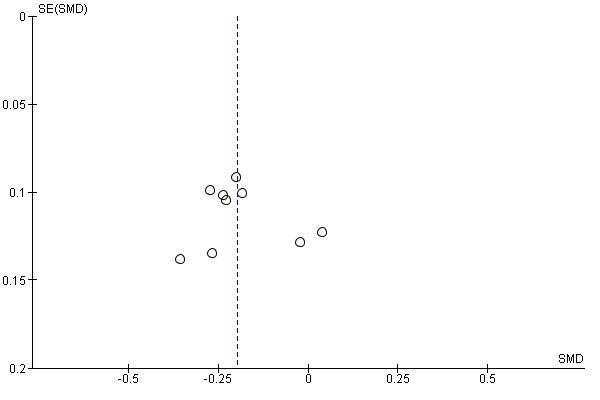
**

**Egger regression test**

p = 0.677

**Figure S4. Discontinuation due to adverse event**

**
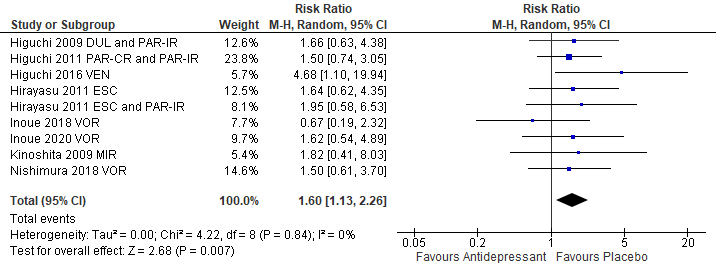
**

**Figure S5. At least one adverse event**

**
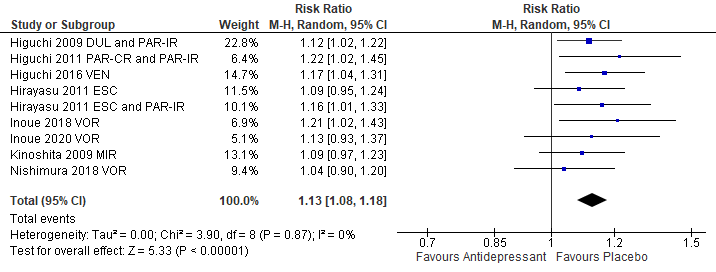
**

**Table S1. The differences in recommended dose of antidepressant between Japan and USA.**

|  | Japan (mg/d) | USA (mg/d) |
| --- | --- | --- |
| Amitriptyline | 30-300 | 50-300 |
| Amoxapine | 25-300 | 100-600 |
| Clomipramine | 50-225 | 25-250 |
| Dosulepin | 75-150 | Dosulepin is not available in USA. |
| Duloxetine | 20-60 | 40-120 |
| Escitalopram | 10-20 | 10-20 |
| Fluvoxamine | 50-150 | Fluvoxamine is approved in USA for OCD. |
| Imipramine | 25-300 | 75-300 |
| Lofepramine | 20-150 | Lofepramine is not available in USA. |
| Maprotiline | 30-75 | 75-225 |
| Mianserin | 30-60 | Mianserin is not available in USA. |
| Milnacipran | 25-100 | Milnacipran is approved in USA for fibromyalgia. |
| Mirtazapine | 15-45 | 15-45 |
| Nortriptyline | 30-150 | 75-150 |
| Paroxetine CR | 12.5-50 | 25-62.5 |
| Paroxetine IR | 10-40 | 20-50 |
| Sertraline | 25-100 | 50-200 |
| Setiptiline | 3-6 | Setiptiline is not available in USA. |
| Trazodone | 75-200 | 150-400 |
| Trimipramine | 50-300 | 75-300 |
| Venlafaxine | 37.5-225 | 37.5-225 |
| Vortioxetine | 10-20 | 10-20 |

CR: controlled-release, IR: immediate release, OCD: obsessive-compulsive disorder, USA: United States of America

**Table S2. Characteristics of the randomized, controlled trials in our systematic review and meta-analysis**

|  | Duration (week) | Diagnosis | %MDD | Minimum severity | PLA lead in | Patient Status | Country | Concomitant Medication | Drug | Dose (mg/d) | Dosing Schedule | n | Age (mean±SD) | %male | Severity at BL (mean±SD) |
| --- | --- | --- | --- | --- | --- | --- | --- | --- | --- | --- | --- | --- | --- | --- | --- |
| Higuchi 2009 DUL vs PAR-IR vs PLA | 6 | DSM-IV | 100 | HAMD17>19 | Yes | IP and OP | Japan | Allow | DUL60 | 60 | Fixed | 74 | 39±10.1 | 56.8 | 20.4±4.1 (HAMD17) |
|  |  |  |  |  |  |  |  |  | DUL40 | 40 | Fixed | 73 | 37.9±9.3 | 52.1 | 20.6±4.4 (HAMD17) |
|  |  |  |  |  |  |  |  |  | PAR-IR | 20-40 | Flexible | 148 | 37.9±9.6 | 54.7 | 20.4±4.8 (HAMD17) |
|  |  |  |  |  |  |  |  |  | PLA |  |  | 145 | 38.7±10.5 | 54.5 | 20.4±4.2 (HAMD17) |
| Higuchi 2011 PAR-CR vs PAR-IR vs PLA | 8 | DSM-IV-TR | 100 | HAMD17>20 | Yes | IP and OP | Japan–South Korea | Allow | PAR-CRL* | 25-50 | Flexible | 79 | NR | NR | NR |
|  |  |  |  |  |  |  |  |  | PAR-IRL† | 20-40 | Flexible | 43 | 35.5±10.38 (PARIR) | 42.2 (PARIR) | 22.7±2.64 (HAMD17) |
|  |  |  |  |  |  |  |  |  | PAR-IRH‡ | 20-40 | Flexible | 40 |  |  |  |
|  |  |  |  |  |  |  |  |  | PLA |  |  | 171 | 36.8±10.07 | 45 | 22.6±2.75 (HAMD17) |
| Higuchi 2016 VEN vs PLA | 8 | DSM-IV-TR | 100 | MADRS>26, QIDS16-SR-J>16, and CGI-S>4 | No | OP | Japan | Allow | VEN75-225 | 75-225 | Flexible | 179 | 38.3±10.2 | 52 | 32.9±4.8 (MADRS) |
|  |  |  |  |  |  |  |  |  | VEN75 | 75 | Fixed | 174 | 38.4±11.9 | 48.3 | 32.6±4.4 (MADRS) |
|  |  |  |  |  |  |  |  |  | PLA |  |  | 184 | 38.6±11.1 | 49.5 | 33.2±5.1 (MADRS) |
| Hirayasu 2011 ESC vs PAR-IR vs PLA | 8 | DSM-IV-TR | 100 | MADRS>22 and CGI-S>4 | Yes | OP | Japan | Allow | ESC20 | 20 | Fixed | 119 | 35.7±9.8 | 42 | 29.8±6 (MADRS) |
|  |  |  |  |  |  |  |  |  | ESC10 | 10 | Fixed | 120 | 36.3±9.7 | 53.3 | 29.4±5.8 (MADRS) |
|  |  |  |  |  |  |  |  |  | PAR-IR | 20-40 | Flexible | 121 | 36.3±8.4 | 45.5 | 29.8±5.9 (MADRS) |
|  |  |  |  |  |  |  |  |  | PLA |  |  | 124 | 36.4±10.8 | 48.4 | 29±5.6 (MADRS) |
| Hirayasu 2011 ESC vs PLA | 8 | DSM-IV-TR | 100 | HAMD17>18 | No | OP | Japan | Allow | ESC20 | 20 | Fixed | 101 | 35.9±11.2 | 56.4 | 22.2±3.7 (HAMD17) |
|  |  |  |  |  |  |  |  |  | ESC10 | 10 | Fixed | 96 | 33.1±9.0 | 37.5 | 22.3±3.5 (HAMD17) |
|  |  |  |  |  |  |  |  |  | PLA |  |  | 100 | 34.7±8.6 | 52 | 22.5±3.6 (HAMD17) |
| Inoue 2018 VOR vs PLA | 8 | DSM-IV-TR | 100 | MADRS>26 and CGI-S>4 | No | NR | Japan | Allow | VOR10 | 10 | Fixed | 123 | 38.8±10.99 | 56.1 | 32.5±4.93 (MADRS) |
|  |  |  |  |  |  |  |  |  | PLA |  |  | 124 | 37.6±10.67 | 46 | 32.5±4.50 (MADRS) |
| Inoue 2020 VOR vs PLA | 8 | DSM-IV-TR | 100 | MADRS>26, HAMD17>18 and CGI-S>4 | Yes | OP | Japan | Allow | VOR20 | 20 | Fixed | 164 | 40.4±11.3 | 51.2 | 30.6±3.6 (MADRS) |
|  |  |  |  |  |  |  |  |  | VOR10 | 10 | Fixed | 165 | 40.0±10.6 | 56.4 | 30.8±3.7 (MADRS) |
|  |  |  |  |  |  |  |  |  | PLA |  |  | 164 | 39.5±10.5 | 56.1 | 30.5±3.9 (MADRS) |
| Kinoshita 2009 MIR vs PLA | 6 | DSM-IV-TR | 100 | HAMD17>18 | No | IP and OP | Japan | Allow | MIR45 | 45 | Fixed | 69 | 41.6±12.5 | 46.4 | 22.1±3.2 (HAMD17) |
|  |  |  |  |  |  |  |  |  | MIR30 | 30 | Fixed | 66 | 37.7±10.8 | 51.5 | 22.5±3.3 (HAMD17) |
|  |  |  |  |  |  |  |  |  | MIR15 | 15 | Fixed | 65 | 39.4±10.9 | 46.2 | 23.2±4.5 (HAMD17) |
|  |  |  |  |  |  |  |  |  | PLA |  |  | 70 | 39.9±12.8 | 55.7 | 22.5±3.6 (HAMD17) |
| Nishimura 2018 VOR vs PLA | 8 | DSM-IV-TR | 100 | MADRS>26 and CGI-S>4 | No | NR | International | Allow | VOR20 | 20 | Fixed | 154 | 44±11.79 | 39.6 | 31.7±3.73 (MADRS) |
|  |  |  |  |  |  |  |  |  | VOR10 | 10 | Fixed | 150 | 45.7±10.9 | 38 | 31.8±4.02 (MADRS) |
|  |  |  |  |  |  |  |  |  | PLA |  |  | 152 | 43.6±11.57 | 40.1 | 31.6±3.56 (MADRS) |

*The starting dose: 12.5 mg

†The starting dose: 10 mg

‡The starting dose: 20 mg

BL: baseline, CGI-S: Clinical Global Impressions-Severity, DSM(-TR): Diagnostic and Statistical Manual of Mental Disorders (-Text Revision), DUL: duloxetine, ESC: escitalopram, HAMD: Hamilton Depression Rating Scale, IP: inpatient, MADRS: Montgomery Åsberg Depression Rating Scale, MDD: major depressive disorder, MIR: mirtazapine, n: number of individuals, NR= not report, OP: outpatient, PAR-CR (-IR): paroxetine-controlled release (-immediate release), PLA: placebo, QIDS16-SR-J: 16-item Quick Inventory of Depressive Symptomatology self-report version, SD: standard deviation, VEN: venlafaxine, VOR: vortioxetine

**Table S3. Efficacy results of the original study and data synthesis of our meta-analysis**

| Study | Depression scale | Results of the study* | The definition of response | Results of the study* | The definition of remission | Results of the study* |
| --- | --- | --- | --- | --- | --- | --- |
| Higuchi 2009 DUL vs PAR-IR vs PLA | HAMD17 (LOCF) | DUL > PLA  PAR-IR = PLA | ≥ 50% improvement in HAMD17 (LOCF) | DUL > PLA  PAR-IR > PLA | HAMD17 < 7 (LOCF) | DUL > PLA  PAR-IR > PLA |
| Higuchi 2011 PAR-CR/PAR-IR vs PLA | HAMD17 (LOCF) | PAR-CR > PLA  PAR-IR > PLA | ≥ 50% improvement in HAMD17 (LOCF) | PAR-CR > PLA  PAR-IR = PLA | HAMD17 < 7 (LOCF) | PAR-CR > PLA  PAR-IR > PLA |
| Higuchi 2016 VEN vs PLA | HAMD17 (LOCF) | VEN75 > PLA  VEN75-225 = PLA | ≥ 50% improvement in HAMD17 (LOCF) | VEN75 = PLA  VEN75-225 = PLA | HAMD17 < 7 (LOCF) | VEN75 > PLA  VEN75-225 = PLA |
| Hirayasu 2011 ESC vs PAR-IR vs PLA | MADRS (LOCF) | ESC > PLA  PAR-IR > PLA | ≥ 50% improvement in MADRS (LOCF) | ESC > PLA  PAR-IR vs PLA (no result) | MADRS < 10 (LOCF) | ESC = PLA  PAR-IR vs PLA (no result) |
| Hirayasu 2011 ESC vs PLA | HAMD17 (LOCF) | ESC10 = PLA  ESC20 = PLA | ≥ 50% improvement in HAMD17 (LOCF) | ESC10 = PLA  ESC20 = PLA | HAMD17 < 7 (LOCF) | ESC10 = PLA  ESC20 = PLA |
| Inoue 2018 VOR vs PLA | MADRS (LOCF) | VOR = PLA | ≥ 50% improvement in MADRS (LOCF) | VOR = PLA | MADRS < 10 (LOCF) | VOR = PLA |
| Inoue 2020 VOR vs PLA | MADRS (LOCF) | VOR10 > PLA  VOR20 > PLA | ≥ 50% improvement in MADRS (LOCF) | VOR10 > PLA  VOR20 > PLA | MADRS < 10 (LOCF) | VOR10 > PLA  VOR20 > PLA |
| Kinoshita 2009 MIR vs PLA | HAMD17 (LOCF) | MIR15 > PLA  MIR30 > PLA  MIR45 = PLA | ≥ 50% improvement in HAMD17 (LOCF) | MIR15 = PLA  MIR30 = PLA  MIR45 = PLA | HAMD17 < 7 (LOCF) | MIR15 = PLA  MIR30 = PLA  MIR45 = PLA |
| Nishimura 2018 VOR vs PLA | MADRS (LOCF) | VOR10 = PLA  VOR20 = PLA | ≥ 50% improvement in MADRS (LOCF) | VOR10 > PLA  VOR20 > PLA | MADRS < 10 (LOCF) | VOR10 = PLA  VOR20 = PLA |

*A = B: A was similar to B, A > B: A was superior to B.

DUL: duloxetine, ESC: escitalopram, HAMD: Hamilton Depression Rating Scale, LOCF: Last Observation Carried Forward, MADRS: Montgomery Åsberg Depression Rating Scale, MIR: mirtazapine, PAR-CR (-IR): paroxetine-controlled release (-immediate release), PLA: placebo, VEN: venlafaxine, VOR: vortioxetine

**Table S4. PRISMA for Pairwise Meta-Analyses Checklist.**

| **Section and Topic** | **Item #** | **Checklist item** | **Location where item is reported** |
| --- | --- | --- | --- |
| **TITLE** | | |  |
| Title | 1 | Identify the report as a systematic review. | P1 |
| **ABSTRACT** | | |  |
| Abstract | 2 | See the PRISMA 2020 for Abstracts checklist. | P3 |
| **INTRODUCTION** | | |  |
| Rationale | 3 | Describe the rationale for the review in the context of existing knowledge. | P4 |
| Objectives | 4 | Provide an explicit statement of the objective(s) or question(s) the review addresses. | P4 |
| **METHODS** | | |  |
| Eligibility criteria | 5 | Specify the inclusion and exclusion criteria for the review and how studies were grouped for the syntheses. | P4 |
| Information sources | 6 | Specify all databases, registers, websites, organisations, reference lists and other sources searched or consulted to identify studies. Specify the date when each source was last searched or consulted. | P4 |
| Search strategy | 7 | Present the full search strategies for all databases, registers and websites, including any filters and limits used. | P4 |
| Selection process | 8 | Specify the methods used to decide whether a study met the inclusion criteria of the review, including how many reviewers screened each record and each report retrieved, whether they worked independently, and if applicable, details of automation tools used in the process. | P4 |
| Data collection process | 9 | Specify the methods used to collect data from reports, including how many reviewers collected data from each report, whether they worked independently, any processes for obtaining or confirming data from study investigators, and if applicable, details of automation tools used in the process. | P4 |
| Data items | 10a | List and define all outcomes for which data were sought. Specify whether all results that were compatible with each outcome domain in each study were sought (e.g. for all measures, time points, analyses), and if not, the methods used to decide which results to collect. | P4 |
|  | 10b | List and define all other variables for which data were sought (e.g. participant and intervention characteristics, funding sources). Describe any assumptions made about any missing or unclear information. | P4 |
| Study risk of bias assessment | 11 | Specify the methods used to assess risk of bias in the included studies, including details of the tool(s) used, how many reviewers assessed each study and whether they worked independently, and if applicable, details of automation tools used in the process. | P4 |
| Effect measures | 12 | Specify for each outcome the effect measure(s) (e.g. risk ratio, mean difference) used in the synthesis or presentation of results. | P4 |
| Synthesis methods | 13a | Describe the processes used to decide which studies were eligible for each synthesis (e.g. tabulating the study intervention characteristics and comparing against the planned groups for each synthesis (item #5)). | P4 |
|  | 13b | Describe any methods required to prepare the data for presentation or synthesis, such as handling of missing summary statistics, or data conversions. | P4 |
|  | 13c | Describe any methods used to tabulate or visually display results of individual studies and syntheses. | P4 |
|  | 13d | Describe any methods used to synthesize results and provide a rationale for the choice(s). If meta-analysis was performed, describe the model(s), method(s) to identify the presence and extent of statistical heterogeneity, and software package(s) used. | P4 |
|  | 13e | Describe any methods used to explore possible causes of heterogeneity among study results (e.g. subgroup analysis, meta-regression). | P4 |
|  | 13f | Describe any sensitivity analyses conducted to assess robustness of the synthesized results. | P4 |
| Reporting bias assessment | 14 | Describe any methods used to assess risk of bias due to missing results in a synthesis (arising from reporting biases). | P4 |
| Certainty assessment | 15 | Describe any methods used to assess certainty (or confidence) in the body of evidence for an outcome. | P4 |
| **RESULTS** | | |  |
| Study selection | 16a | Describe the results of the search and selection process, from the number of records identified in the search to the number of studies included in the review, ideally using a flow diagram. | P6 |
|  | 16b | Cite studies that might appear to meet the inclusion criteria, but which were excluded, and explain why they were excluded. | P6 |
| Study characteristics | 17 | Cite each included study and present its characteristics. | P6 |
| Risk of bias in studies | 18 | Present assessments of risk of bias for each included study. | P6 |
| Results of individual studies | 19 | For all outcomes, present, for each study: (a) summary statistics for each group (where appropriate) and (b) an effect estimate and its precision (e.g. confidence/credible interval), ideally using structured tables or plots. | P6 |
| Results of syntheses | 20a | For each synthesis, briefly summarise the characteristics and risk of bias among contributing studies. | P6 |
|  | 20b | Present results of all statistical syntheses conducted. If meta-analysis was done, present for each the summary estimate and its precision (e.g. confidence/credible interval) and measures of statistical heterogeneity. If comparing groups, describe the direction of the effect. | P6 |
|  | 20c | Present results of all investigations of possible causes of heterogeneity among study results. | P6 |
|  | 20d | Present results of all sensitivity analyses conducted to assess the robustness of the synthesized results. | P7 |
| Reporting biases | 21 | Present assessments of risk of bias due to missing results (arising from reporting biases) for each synthesis assessed. | P6 |
| Certainty of evidence | 22 | Present assessments of certainty (or confidence) in the body of evidence for each outcome assessed. | P6 |
| **DISCUSSION** | | |  |
| Discussion | 23a | Provide a general interpretation of the results in the context of other evidence. | P7 |
|  | 23b | Discuss any limitations of the evidence included in the review. | P7 |
|  | 23c | Discuss any limitations of the review processes used. | P7 |
|  | 23d | Discuss implications of the results for practice, policy, and future research. | P7 |
| **OTHER INFORMATION** | | |  |
| Registration and protocol | 24a | Provide registration information for the review, including register name and registration number, or state that the review was not registered. | P4 |
|  | 24b | Indicate where the review protocol can be accessed, or state that a protocol was not prepared. | P4 |
|  | 24c | Describe and explain any amendments to information provided at registration or in the protocol. | P4 |
| Support | 25 | Describe sources of financial or non-financial support for the review, and the role of the funders or sponsors in the review. | P8 |
| Competing interests | 26 | Declare any competing interests of review authors. | P8 |
| Availability of data, code and other materials | 27 | Report which of the following are publicly available and where they can be found: template data collection forms; data extracted from included studies; data used for all analyses; analytic code; any other materials used in the review. | P8 |

*From:*  Page MJ, McKenzie JE, Bossuyt PM, Boutron I, Hoffmann TC, Mulrow CD, et al. The PRISMA 2020 statement: an updated guideline for reporting systematic reviews. BMJ 2021;372:n71. doi: 10.1136/bmj.n71

For more information, visit: <http://www.prisma-statement.org/>
